# New advances in the treatment of thin endometrium

**DOI:** 10.3389/fendo.2024.1269382

**Published:** 2024-04-30

**Authors:** Yidi Wang, Zunhao Tang, Xiuxiang Teng

**Affiliations:** ^1^ Beijing Hospital of Traditional Chinese Medicine, Capital Medical University, Beijing, China; ^2^ College of Traditional Chinese Medicine, Shandong University of Traditional Chinese Medicine, Jinan, China

**Keywords:** thin endometrium, estrogen, stem cells, platelet-rich plasma, endometrial blood perfusion

## Abstract

Thin endometrium (TE) is defined as a mid-luteal endometrial thickness ≤7mm. TE can affect endometrial tolerance, leading to lower embryo implantation rates and clinical pregnancy rates, and is also associated with impaired outcomes from assisted reproductive treatment. Herein, we systematically review TE causes, mechanisms, and treatments. TE pathogenesis has multiple causes, with the endometrium becoming thinner with age under hormonal influence. In addition, uterine cavity factors are important, as the inflammatory environment may affect expressions of certain genes thereby inhibiting endometrial stromal cell proliferation and promoting apoptosis. Long-term oral contraceptive use or the use of ovulation-promoting drugs are also definite factors contributing to endometrial thinning. Other patients have primary factors, for which the clinical etiology remains unknown. The main therapeutic strategies available for TE are pharmacological (including hormonal and vasoactive drugs), regenerative medicine, intrauterine infusion of growth factor-granulocyte colony-stimulating factor, autologous platelet-rich plasma, and complementary alternative therapies (including traditional Chinese herbal medicine and acupuncture). However, the associated mechanisms of action are currently unclear. Clinical scholars have proposed various approaches to improve treatment outcomes in patients with TE, and are exploring the principles of efficacy, offering potentials for novel treatments. It is hoped that this will improve TE tolerance, increase embryo implantation rates, and help more couples with infertility with effective treatments.

## 1 Introduction

Embryo implantation success depends mainly on two conditions: the degree of embryo development and endometrial receptivity (ER). ER refers to the ability of the endometrium to allow the embryo to contact, localize, adhere, and implant at a specific time, referred to as the *window of implantation* (WOI) (1). The WOI, usually days 20–24 of the normal menstrual cycle or days 7–10 of the LH peak, is when the embryo is most likely to implant. Ultrasonography is used as a routine endometrial assessment method, in which endometrial thickness (EMT) is an important parameter for assessing endometrial tolerance, which is critical to successful pregnancy (2). While there are not currently standardized, clear diagnostic criteria for thin endometrium (TE), <7mm is both the most common factor of uterine infertility and is associated with impaired outcomes of assisted reproductive treatments. Globally, 8–12% of couples of reproductive age are affected by infertility, and assisted reproductive technology (ART) can solve most infertility causes (e.g., ovulatory dysfunction, male factor infertility, tubal disease). As use of ART has expanded, endometrial tolerance is widely recognized as a key factor in its success. TE leads to lower implantation rates (IRs), increased obstetric complications, and adverse perinatal outcomes. Previous studies have shown a negative correlation between EMT and the incidence of preterm birth, and that EMT of 7.8mm is an independent predictor of higher preterm birth following fresh embryo transfer (3). Other studies have found that EMT is associated with a decrease in clinical pregnancy rate (CPR) and live birth rate (LBR) when <8mm at fresh embryo transfer or <7mm at frozen-thawed embryo transfer (FET). In contrast, LBR increases with EMT at fresh embryo transfer, and plateaus when EMT reaches 10–12mm into the plateau; in FET cycles, LBR tends to stabilize after EMT increases to 7–10mm (4, 5). The mechanisms by which TE leads to poor endometrial tolerance remain to be further explored, and a variety of methods have been used clinically to improve outcomes in patients with TE, such as estrogen therapy, low-dose aspirin, sildenafil citrate, vitamin E, regenerative medicine therapies, and complementary and alternative therapies. This review of the literature related to TE treatment analyzes the studies on improving EMT and tolerance, with the aim of providing a more comprehensive understanding of this disease and its treatment modalities, and highlighting potentials for exploring for new therapeutic ideas.

## 2 TE definition

In most cases, ultrasonography defines TE as ≤7mm (6); however, the definition of ‘thin’ varies from ≤6–8mm (7–10). TE prevalence increases with age, reaching 25% in natural cycles among women over age 40 years. TE incidence in infertility treatment cycles is 2–3% (11). In ART, endometrial tolerance and embryo quality are two main factors affecting *in vitro* fertilization (IVF) and embryo transfer cycles, with nearly two-thirds of implantation failures in IVF reported to be due to reduced endometrial tolerance, and the remaining one-third attributed to embryo quality (12).

## 3 Causes and mechanisms of endometrial thinning

The many causes of decreased EMT are traditionally categorized as inflammatory, medical, and idiopathic (13). Anatomically, acquired uterine abnormalities like uterine adhesions, endometrial polyps, endometriosis, fibroids, and other uterine abnormalities may disrupt endometrial environmental homeostasis, leading to diminished endometrial tolerance (14). For example, chronic endometritis may affect the endometrial immune milieu by destroying the associated immune cells. Studies have found impairments in metabolic signaling pathways in thin endometrial tissues, and coordinated estrogen–progesterone interactions among multiple cells are critical for uterine tolerance. Anatomical, immunological, and metabolic factors also influence endometrial tolerance, and more clinical large-scale randomized controlled studies are needed to address these. TE is characterized by poor glandular epithelial growth, high impedance to uterine blood flow, reduced vascular endothelial growth factor (VEGF) expression, and poor vascularization compared with normal-thickness endometrium (15).

## 4 Dangers of endometrial thinning

Although the etiologies of endometrial thinning vary, they lead to similar outcomes: reduced embryo implantation and pregnancy rates, and increased miscarriage risks. TE has been associated with a higher incidence of early miscarriage (i.e., miscarriage and ectopic pregnancy), intrauterine growth restriction, and a composite adverse perinatal outcome (16, 17). Infants of patients with TE also have a twofold increased risk of low birth weight and preterm labor (18).

## 5 TE treatments

### 5.1 Drug therapies

#### 5.1.1 Endocrine therapies

##### 5.1.1.1 Estrogen

Estrogen secretion increases during the follicular phase of the normal menstrual cycle and binds to estrogen receptors to promote proliferation and regeneration of endometrial epithelial cells (EECs). Estrogen affects embryo implantation via endometrial estrogen receptor α and cytokines such as leukocyte inhibitory factor (LIF) and IL-6 (19–22); thus, it can also positively affect endometrial tolerance.

The concentration, duration of action, and route of supplementation of estrogen may also be related to endometrial tolerance. A retrospective study by Amir et al. found that EMT correlates with serum estradiol levels up to 1000 pg/ml, and that the correlation was not significant >1000 pg/ml (23). It has also been reported that endometrial growth correlates with duration of estrogen action, while there is no significant correlation with estrogen levels (24).

Estrogen supplementation can be administered in several ways, among which the oral route is more convenient and transdermal ingestion has the most pronounced effects. A prospective randomized controlled study by Yi et al. found similar endometrial growth in patients with uterine adhesions after one cycle of oral estradiol valerate tablets versus transdermal gel treatment after hysteroscopy (25). The highest serum drug concentrations and endometrial proliferation rates are obtained with transvaginal estrogen administration, which is recommended after other modes have failed (26). Regarding dosage, patients with TE are not sensitive enough to respond to normal physiologic doses of estrogen because of their relatively low number, and dysfunction, of estrogen receptors. However, when using estrogen to treat TE, the risk of endometrial pathology remains an important consideration (e.g., endometrial cancer, thrombosis). These may result from prolonged, high-dose estrogen use, and the endometrium should also be protected by regular progestin use, except for contraindications. In conclusion, the role of estrogen in promoting endothelial growth remains controversial, and the administration mode, usage, and dosage require further standardization.

##### 5.1.1.2 Growth hormone

GH is a peptide hormone secreted by the pituitary gland, the receptors for which are found on the human endometrium. GH improves EMT, and possibly endometrial tolerance, in women with infertility, possibly by mechanisms such as increased endometrial blood perfusion, increased expression of genes and proteins associated with endometrial tolerance, and induction of insulin-like growth factor 1 (IGF-1) production; however, the exact mechanisms remain to be elucidated (27, 28).

The effectiveness of GH treatment on endometrial quality and function in women undergoing IVF was evaluated in a systematic review and meta-analysis (29). Adjuvant application of GH was found to not only increase the LBR, CPR, and IR, it also improved EMT. Based on subgroup analysis, a dose- and time-dependent relation between GH combination therapy and CPR was also found. Thus, GH may promote fertility by improving endometrial quality.

##### 5.1.1.3 Human chorionic gonadotropin

HCG is a glycoprotein hormone synthesized by gestational trophoblasts and is commonly used as a trigger drug in ovulation promotion. HCG receptor expression is also present on the endometrium during the endometrial secretory phase. Beyond a certain concentration, HCG instilled in the uterine cavity before embryo transfer can bind to receptors to upregulate expression factors including VEGF, LIF, matrix metalloproteinase-9 (MMP-9) (30); these cytokines improve maternal–fetal immune tolerance, upregulating endometrial thickness, and improving pregnancy outcomes.

##### 5.1.1.4 Gonadotropin-releasing hormone agonist

GnRH-a drugs are the first-line of clinical use in assisted reproduction and endometriosis. After the first application of GnRH-a, it binds to the GnRH receptor in the pituitary gland and instantly stimulates the release of gonadotropins (follicle stimulating hormone and luteinizing hormone) from pituitary cells, resulting in a ‘flare-up’ effect, which raises the serum estrogen level and stimulates the body to produce the cytokines necessary for implantation, thus improving the EMT and increasing endometrial tolerance (31). GnRH-a in combination with human menopausal gonadotropin also enhances the stimulation of follicular development and improves EMT in women undergoing FET after intensified estrogen administration (32). A retrospective study also found that clinical pregnancy and LBRs were significantly higher in the GnRH agonist group than in the GnRH antagonist group (33).

##### 5.1.1.5 Tamoxifen

TMX is a selective estrogen receptor modulator. As an antitumor drug, it has both anti-estrogenic and estrogen-like effects, and plays different roles in different target organs, inhibiting breast tumors while promoting endometrial hyperplasia. Combination TMX+hormone replacement therapy (HRT) in patients with infertility with TE (EMT <8mm) increased EMT more than HRT alone; however, differences in clinical pregnancy outcomes (CPR, embryo IR, early miscarriage rate, LBR) did not differ significant between these groups (34).

Clinical studies have found that TMX somewhat improves EMT and LBR and reduces the likelihood of multiple pregnancies in patients with TE who are treated with clomiphene; however, in patients with polycystic ovary syndrome, the ovulation induction by TMX may be inadequate (35). It is also clear that current use of TMX in reproduction is beyond its scope of application, and its safety remains unclear.

#### 5.1.2 Increased endometrial blood perfusion

##### 5.1.2.1 Low-dose aspirin

Aspirin, a cyclooxygenase inhibitor, does not directly increase EMT, but may improve pregnancy outcomes by improving endometrial morphology, perfusion, and tolerance (36). In a prospective randomized study of aspirin in patients with TE (≤8mm) undergoing intrauterine insemination, aspirin administration improved pregnancy rates and endometrial state; however, it did not significantly increase EMT or improve resistance to uterine and ovarian blood flow (37). Another study showed that supplemental low-dose aspirin after adhesive separation of the uterine cavity for TE may promote endometrial growth and repair, and the combination of low-dose aspirin in patients treated with high-dose estrogens may also help reduce the risk of blood clots (38).

##### 5.1.2.2 Sildenafil citrate

Sildenafil citrate is a specific phosphodiesterase type 5 inhibitor that enhances the vasodilatory effect of nitric oxide on vascular smooth muscle by preventing cGMP degradation. It has been suggested that endometrial thinning may be caused by impedance to high blood flow in uterine arteries, and that vaginal administration of sildenafil citrate may be effective for increasing EMT and CPRs, and improving biochemical pregnancy rates in women with TE (39). A randomized, placebo-controlled trial examining the effect of vaginally administered sildenafil in patients with at least two failed IVF/intracytoplasmic monospermous injection rounds showed that women receiving sildenafil (alone or in combination with placebo) showed a twofold increase in chemical pregnancies compared with the placebo group. This increase was clinically significant, although not statistically significant based on sample size (40).

##### 5.1.2.3 Botulinum toxin A

BoTA is widely used in plastic, reconstructive, and aesthetic surgeries (41, 42). It has also been reported to enhance re-epithelialization of human keratinocytes and angiogenesis of endothelial cells (43). Animal experiments by Koo et al. demonstrated that intrauterine BoTA administration significantly induces endometrial angiogenesis and can enhance endometrial tolerance (44). Further studies found that intrauterine BoTA injection improved the endometrial environment in mice with TE by increasing endometrial tolerance and angiogenesis via the regulatory effect of IGFBP3 on hydrolytic cleavage of OPN proteins; these findings suggest BoTA as a potential therapeutic strategy for patients with TE. However, there is currently insufficient basis for clinical trials, and clinical validation is needed (45).

##### 5.1.2.4 Vitamin E

Vitamin E is a fat-soluble vitamin, the hydrolysis product of which is tocopherol, which increases estrogen levels and improves fertility. Tocopherol is an antioxidant that acts to limit reactive oxygen species (ROS) during oxidative stress. Vitamin E also reduces free radical-induced chromosome damage by inhibiting ROS formation and nucleic acid endonuclease activation. Pentoxifylline (PTX) is a methylxanthine derivative used in the treatment of vascular diseases; it is an antioxidant and promotes vasodilation. In a retrospective study of 143 patients with EMT <7mm and repeated implantation failures, combination tocopherol and PTX treatment significantly improved endometrial volume (EV) thresholds and EMT (46).

### 5.2 Stem cells and their derivatives

Stem cell therapy is a recognized potential therapeutic strategy for endometrial repair and regeneration, due to the potential for stem cells to replace damaged endometrial cells and regenerate damaged endometrial tissue. Many stem cell types have been associated with endometrial renewal and regeneration, and can be applied to endometrial damage repair. According to their differentiation potentials, stem cells are mainly classified as totipotent, pluripotent, multipotent, or unipotent (specialized). Pluripotent stem cells can be renewed and differentiated into many different cell types. Mesenchymal stem cells (MCSs) and hematopoietic stem cells, which are currently in wide use, are multipotent stem cells.

#### 5.2.1 MSCs

MSCs, the most widely studied stem cells, have high self-renewal capacity and multilineage differentiation potential. MSCs derived from bone marrow are known as bone marrow-derived MSCs (BMDMSCs), those obtained and isolated from subcutaneous adipose tissue (e.g., from human thighs, abdominal wall) are known as adipose-derived stem cells (ADSCs), and adult stem cells isolated from menstruation tissues are known as menstrual-derived stem cells (MenSCs). Other types include umbilical cord-derived MSCs (UC-MSCs) and endometrial MSCs.

##### 5.2.1.1 BMDMSCs

Many previous studies have confirmed the positive effects of BMDMSCs on endometrial diseases (47, 48). Cervello et al. found that BMDMSCs accumulated around small blood vessels in the injured endometrium and, through a paracrine manner, up-regulated platelet-responsive protein 1 and downregulated IGF-1 through paracrine means, ultimately leading to proliferation of epithelial glandular cells and thus endometrial thickening (49). By transplanting rat BMDMSCs, Wang et al. were able to perform endometrial repair on model heat-damaged endometrial injury, showing that BMDMSCs helped restore normal endometrial tissue structure, improved fertility, reduced the area of endometrial fibrosis, and improved endometrial tolerance (50).

Patel et al. (48) reported the case of a patient with TE who, despite several attempts at HRT treatment, had suboptimal EMT and failed subsequent IVF. This patient subsequently received BMDMSC therapy via uterine artery injections with interventional radiology, followed by HRT for 3 months, to improve the endometrium. This was followed by donor egg IVF, with EMT 7.1mm achieved before embryo transfer. Treatment outcome was successful conception and delivery of a 3.1 kg male infant without prenatal, intrapartum, or postpartum complications. In a prospective study of 36 patients, Natalya et al. showed significant EMT improvements in patients with TE who were treated with autologous BMDMSCs, demonstrating a good safety profile (51). Though current research favors BMDMSCs as a novel option for patients with Asherman syndrome and recalcitrant TE, it is worth noting that BMDMSC therapy requires bone marrow biopsy and subsequent cell sorting, as well as interventional radiation to gain small uterine artery access; these and other factors make this treatment expensive and potentially harmful. The differentiation ability of BMDMSCs is also related to patient age; thus, more research is needed to guide clinical practice and potential applications of this therapy.

##### 5.2.1.2 MenSCs

MenSCs have multi-directional differentiation abilities. Due to the noninvasive process of obtaining them, the use of MenSCs to treat endometrial pathology has gradually attracted attention in recent years. One researcher collected menstruation tissue from patients with severe uterine cavity adhesions, cultured them *in vitro* to isolate these stem cells, and then reintroduced them into the uterine cavity. Seven cases were successfully isolated and reintroduced, and endometrial thickening to 7mm was observed in five of them; among these, one patient was naturally impregnated and four underwent subsequent frozen embryo transplantation, two of whom were successfully impregnated (52). This study suggests that MenSCs are helpful in the treatment of TE.

Further studies have investigated the mechanism of MenSCs in the treatment of TE and found that these cells stimulate the invasion and proliferation of EECs and regulate cytokines (53). Another animal experiment confirmed that human MenSCs differentiated into endometrial cells *in vitro* can rebuild endometrial tissue after induction with estrogen and progesterone in mice (54). Recently, researchers have converted MenSCs into decidualized stromal cells (DSCs), which are more secretory, and have demonstrated that DSCs promote the restoration of EMT and contribute to the production of glands and blood vessels (55). Currently, MenSC research focuses on animal experiments, but since MenSCs are derived from an individual’s own menstruation tissue, and their production is closely related to the endometrium, clinical application scenarios are worthy of further study. The limitation of this approach is mainly related to difficulty with implementation among amenorrheic women.

##### 5.2.1.3 Adipose-derived stem cells and adipose-derived regenerative cells

ADSCs can be obtained and isolated from subcutaneous adipose tissue (e.g., from human thighs, abdominal wall). ADSCs have the ability of multidirectional differentiation, are relatively available and easy to obtain, have high proliferative activity in *in vitro* cultures, and have been applied to wound repair and skin tissue regeneration. Adipose-derived stromal vascular fraction (AD-SVF) was obtained by enzyme preparation and centrifugation, which contains adipose-derived MSCs, adipose stromal vasculature, and other components. Lee et al. extracted adipose tissue from one patient with severe Asherman syndrome, then injected their AD-SVF through the cervix into the patients’ uterus with subsequent hormone supplementation therapy, eventually observing a significant increase in EMT (56).

ADRCs were obtained by subcutaneous fat isolation and characterized by flow cytometry. Yotsumoto et al. found that ADRCs improved EMT, endometrial fibrosis, and embryo implantation, and increased VEGF expression in the mouse uterus (57). ADRCs may thus become an effective therapeutic strategy to improve fertility in women with TE.

Despite the additional cosmetic effects of liposuction, the overall cost of this treatment remains high, and its clinical efficacy has yet to be validated in trials with larger samples.

##### 5.2.1.4 UC-MSCs

UC-MSCs are derived from discarded neonatal umbilical cord tissue, which are easy to culture *in vitro*, have the ability of multidirectional differentiation, and have been widely used in stem cell transplantation research because of their low immunogenicity, short proliferation cycle, and long survival time. Zhang et al. treated endometrial injury with single and multiple transplants of human UC-MSCs, finding that human UC-MSCs repaired damaged rat endometrium and improved fertility (58). Furthermore, UC-MSCs have been shown to restore EMT, attenuate endometrial injury-induced hyper fibrosis, promote blood vessel growth and endothelial cell proliferation, downregulate some pro-inflammatory factors, and upregulate some anti-inflammatory factors, which may help to restore uterine structure and function. Despite the advantages of this therapy, its clinical application remains controversial due to its allogenic origin. In a study of patients with uterine adhesions who underwent implantation of UC-MSCs and subsequent estrogen-progestin replacement therapy, endometrial thickening and improvement in uterine adhesions were found after three months, and DNA short tandem repeat analysis showed that no xenogenic nucleic acids were detected in the regenerated endometrial tissue (59).

##### 5.2.1.5 Endometrial MSCs

Endometrial MCSs are thought to exhibit similar properties to BMDMSCs and are multipotent in their differentiation, with the ability to differentiate into fat, bone, cartilage, skeletal muscle, and smooth muscle (60, 61). Sapozhak et al. recently reported a case of autologous endometrial MSCs in a patient with TE and a history of previous miscarriages, which resulted in a live birth (62). After endometrial MSCs and subsequent hormonal supplementation, the patient’s EMT grew to 6.9mm; at the patient’s insistence, an embryo transfer was performed, and two weeks after the transfer her HCG was 4,795 IU/L. Four weeks after the transfer, an ultrasound confirmed a bi-chorionic twin pregnancy, which eventually resulted in the birth of a female infant. This study demonstrates that autologous endometrial MSCs can be effective for assisting in promoting EMT growth, and enabling live birth. However, as this is an isolated case, more clinical validation is needed.

#### 5.2.2 Human embryonic stem cells

Human embryonic stem cells are a class of pluripotent cells. They originate from the cell mass within the blastocyst during embryonic development. Among the many stem cell types, these have the most comprehensive differentiation potential. Animal experiments have shown that when endometrial-like cells differentiated from human embryonic stem cells were inoculated into mice with endometrial damage, differentiated cells survived in the mouse uterus and the endometrial damage was repaired (63). Another animal experiment showed that some embryonic stem cells in the uterine cavity of mice after transplantation could form tumor-like swelling, and further observation of the uterus transplanted tumor-like tissue can be seen in cartilage, fat, and other tissues, suggesting potential tumor formation (64).

In sum, stem cell therapy is gradually being explored as a newer therapeutic mode if TE treatment. Cellular and animal experiments to date have shown better prospects for application, but clinical case reports and clinical trials with smaller samples are still insufficient to provide high-level evidence to support the efficacy and safety of this therapy. Additionally, there remain challenges to using stem cells for clinical treatment. For example, harvesting adult stem cells is painful and invasive, and cell expansion is expensive and time-consuming. In addition, endometrial stem cells have the potential to differentiate into endometrial pathologies like endometrial cancer and adenomyosis. The developmental risk of teratomas must also be considered with embryonic stem cell uses. The risk of immune rejection and other ethical issues clearly need to be carefully considered.

#### 5.2.3 Extracellular vesicles and exosomes

EVs are membrane-bound vesicles found in biological fluids, which carry and transfer regulatory molecules such as microRNA and proteins, and may mediate intercellular communication between cells and tissues (65). According to the production mode, release pathway, content, and size of EVs, they can be divided into three categories: Exo, microvesicles (MVs), and apoptotic bodies. Exo are particles with a diameter of 40–60nm that originate from the multivesicular body. MVs have a diameter of 100–1000nm and are formed by the detachment of cell membranes. Apoptotic bodies, with a diameter of 1~5μm, are bubble-shaped bodies formed by the collapse of the cell membrane during programmed cell death, containing DNA material and organelles (66). EVs are produced in different tissues or cells such as endometrium, decidua, embryos, semen, and fallopian tubes during implantation windows. Increasing evidence suggests that EVs are important mediators of intercellular communication, so they play an important role in stem cell support, tissue repair, and immune regulation (67–69). Currently, more studies have focused on Exo, and it has been found that Exo play an important role in acrosome reaction, sperm capacitation, and embryo implantation.

Studies have shown that Exo can participate in cell regulation and physiological and pathological processes in a variety of ways (70). Stem cell-derived EVs/Exo can improve ER in certain pathological conditions. Among the various experiments carried out in this regard (71), Exo from adipose MCSs can improve EMT and glands, and increase pregnancy rates (72). Umbilical cord blood-derived mesenchymal stem cell Exo have been shown to promote epithelial repair and new blood vessel formation, improve endometrial proliferation, improve EMT and glands, and increase pregnancy rate (73, 74). Bone marrow mesenchymal stem cell-derived Exo can also promote endometrial repair (75). MVs secreted by amniotic MCSs have been shown to improve endometrial cell proliferation and anti-apoptosis in *in vitro* experiments (76). Human endometrial MSCs-derived EVs can promote endometrial angiogenesis, differentiation, and tissue remodeling (77). In addition, novel tissue engineering methods are also being explored, such as combining mesenchymal stem cell Exo with collagen scaffold to form a load, allowing Exo to be slowly released at the corresponding site to exert their effects (78). The combination of exosomes with collagen scaffolds enables structural and functional reconstruction of the endometrium to support implantation and development of embryos *in vivo*.

Current stem cell therapy limitations include the abovementioned immune problems, ethical issues, tumorigenicity, and the low retention rate of stem cells at the transplant site caused by the shape of the uterus. As a cell-free therapy strategy instead of live cell transplantation, the EVs/Exo of MSCs have relatively higher biological stability and more easily penetrate tissues.

### 5.3 Intrauterine growth factor-granulocyte colony-stimulating factor infusion

G-CSF is a glycoprotein that acts primarily on neutrophils to promote their value-addition, differentiation, and activation. In the field of reproduction, G-CSF has been found to be involved in follicular growth and development, ovulation, and pregnancy, and has a bidirectional regulatory role in maternal–embryonic exchange.

G-CSF significantly increases EMT in TE rats and may improve endometrial tolerance by stimulating endometrial proliferation and neovascularization (79, 80).

In terms of clinical studies, a study by Gleicher found that four patients with TE previously refractory to estrogen and vasodilator therapy had endometrium that was successfully dilated to at least a minimum thickness of 7 mm after G-CSF infusion, and all four patients also became pregnant (81). Lian found, in a retrospective cohort study of 271 infertile patients with TE, that G-CSF intrauterine infusion could significantly increase EMT in a patient population (82).

However, a retrospective study conducted by Jiang found that when G-CSF was used in group 1 via intracavernous infusion and GH was injected subcutaneously in group 2, the EMT was thinner in the G-CSF group than in the GH group (83).

Overall G-CSF has a favorable effect on endometrial thickening, but the overall sample size of clinical studies is small and larger trials are still needed to validate this.

### 5.4 Autologous platelet-rich plasma

PRP, defined as “the plasma component of autologous blood in which the platelet concentration is four to five times the normal level”, is obtained by centrifugation of autologous peripheral venous blood. Currently, the preparation of second-generation PRP has replaced first-generation PRP (84). Second-generation PRP, also known as leukocyte-rich platelet fibrin, differs from first-generation PRP in that it contains a different proportion of cellular components and a different fibrin architecture, and therefore exerts a higher biological effect, is richer in fibrinogen, and has a longer-lasting effect (85). PRP was originally studied in trauma repair in the 1970s (86), and has been widely used in plastic surgery, including for traumatic scar repair, hair regrowth, and free skin grafting, and in dermatology for the treatment of alopecia and difficult-to-heal wounds (87–89). PRP’s medical value and biosafety have been clinically recognized and verified, but its application in obstetrics and gynecology is still at the beginning stage. Most current studies show that PRP can improve pregnancy outcomes in TE, and a small number of studies suggest that PRP’s effect in improving TE is non-obvious. The common administration methods include drip injection at endometrial group nodes under hysteroscopy, injection of the drug into the endometrium through an endoscopic needle under hysteroscopic visualization, and drip/perfusion through a uterine catheter. The number of injections varied from 1–3 and time spacing.

Results from a prospective cohort study showed that transuterine catheter injection of PRP on day 10 of the HRT cycle, and on the day of progesterone administration are both effective for improving EMT, increasing CPR and embryo IR, and reducing cycle cancellation rate (90). Other retrospective and open-label randomized controlled studies support the conclusion that PRP increases EMT, and improves pregnancy and LBRs after intrauterine instillation (91, 92).

In an *in vitro* test by Yang et al. on the effects of PRP on the proliferation of endometrial mesenchymal cells, the content of platelet factor PDGF-AB in umbilical cord venous blood of neonates at 37–41 weeks was higher than that in peripheral venous blood of pregnant women at the same gestational week. They also showed that low concentrations of PRP could stimulate cell proliferation and migration in a dose-dependent pattern, and that over a certain concentration range cell proliferation was inhibited and was strongest in the case of the 2% PRP dosage (93, 94). Therefore, the dose concentration of PRP is closely related to its clinical efficacy, suggesting that it is clinically significant to find the most suitable concentration of PRP applied for uterine perfusion.

In terms of possible mechanisms, PRP plays a key role in cell proliferation, regeneration, and differentiation because it contains various growth factors. These growth factors, such as VEGF, PDGF, EGF, TGF, IGF1, and other cytokines, are released by platelet activation at sites of injury or inflammation. Among these growth factors, VEGF is responsible for vascularization. PRP stimulates the proliferation of different endometrial cells, including epithelial cells, endometrial stromal fibroblasts, and endometrial mesenchymal stem/progenitor cells. In experiments, the addition of PRP significantly increased cell proliferation in the human endometrial stromal cell line (i.e., ICE7). Similarly, a replicative effect was observed when primary stromal cells were used. Furthermore, after PRP application, higher levels of Hoxa10, a major endometrial tolerance marker, were detected in the endometrium. This indicates the potential impact of PRP on endometrial tolerance.

### 5.5 Complementary and alternative therapies

A meta-study demonstrated that Jin Feng Wan is effective in improving the clinical symptoms of thin endometrial infertility, increasing pregnancy rate, decreasing miscarriage rate, and improving EMT; however, a small number of articles was included, their quality was poor, and the level of evidence was low (95). Ding Kun Dan in combination with estradiol valerate improves endometrial tolerance by up-regulating β-linker protein and VEGF mRNA expression and down-regulating MMP-9 mRNA expression in rats with endometrial injury (96). An animal study by Yin et al. showed that a combination regimen of kidney tonic, blood-boosting tonic, and estradiol valerate significantly improved the pregnancy rates in female rats with TE and ovulation disorders, and in female rats with thin endometrial ovulation disorders (97). The treatment was accompanied by a significant increase in EMT and a decrease in the uterine artery pulsatility index (PI) and endometrial resistance index (RI).

The effects of acupuncture on endometrial tolerance and thickness have been confirmed by a randomized controlled trial (98). This study showed that endovascular therapy combined with acupuncture significantly increased EMT and decreased bilateral uterine artery PI, uterine RI, and peak systolic/diastolic velocities. It also up-regulated the HOXA10 protein expression and mRNA in endometrial tissues, and increased the rate of embryo implantation and clinical pregnancy in patients with TE. This may be related to AMPK/mTOR signaling pathway modulation, down-regulation of AMPK gene and protein expression, and up-regulation of mTOR gene and protein expression by intracavitary physiotherapy combined with acupuncture.

Massage also improves EMT and blood flow. Shen et al. randomized patients prepared for FET into a study or control group. Hormone supplementation was used in both groups, and pelvic floor muscle massage was added to the treatment group (99); the treatment group experienced improved EMT and sub-endometrial blood flow, which in turn improved pregnancy rates in that group.

The detailed effects of various treatment methods are described in **Table 1**.

**Table 1:** Treatment of thin endometrium in different ways and their effects.

Treatment		Drug	Study design	Function	Reference
Estrogen		Estradiol valerate	Case report, retrospective study, prospective randomized controlled study.	Estradiol valerate can increase endometrial thickness, improve clinical pregnancy rate and embryo transfer rate. In a certain range, estrogen level is correlated with endometrial growth, and estrogen action time is also correlated with endometrial growth. Different routes of administration have different effects.	(22–26)
Growth hormone		RhGH subcutaneous injection, GH intrauterine injection.	Prospective study, Meta analysis.	Simultaneous administration of RhGH and HRT can improve clinical efficacy. Intrauterine injection of GH is more targeted and can effectively increase endometrial thickness. Meta-analysis found that adjuvant GH can increase live birth rate, clinical pregnancy rate and implantation rate, and improve endometrial thickness.	(27–29)
Human chorionic gonadotropin (HCG)		HCG	Vitro molecular biology research.	Intrauterine infusion of HCG at a certain concentration before embryo transfer can improve the immune tolerance between mother and embryo, thereby increasing the endometrial thickness and pregnancy outcome.	(30)
Gonadotropin-releasing hormone agonist (GnRH-a)		GnRH-a	Retrospective study.	GnRH-a can improve endometrial thickness, enhance endometrial receptivity, and increase clinical pregnancy rate and live birth rate.	(32, 33)
Tamoxifen (TMX)		TMX	Retrospective study, prospective study.	TMX adjuvant application can increase endometrial thickness, but there is no significant difference in pregnancy outcome. Prospective studies have shown higher pregnancy and live birth rates in the TMX group.	(34, 35)
Aspirin		Low-dose aspirin	Prospective cohort study.	Promote the growth and repair of intima and reduce the risk of thrombosis.	(37, 38)
Sildenafil citrate		Sildenafil citrate	RCT, Meta analysis.	Increase endometrial thickness and improve clinical pregnancy rate.	(39, 40)
Botulinum toxin A		Botulinum toxin A	*In vivo* study.	Improve endometrial receptivity, but still need clinical validation.	(44)
Vitamin E		Vitamin E	Retrospective study.	Improve endometrial receptivity.	(46)
Stem cell therapies	Mesenchymal stem cells	Bone marrow mesenchymal stem cells	Case report, experiment on rats, prospective studies.	It can help repair thin endometrium and increase endometrium thickness. It can be expensive.	(48–51)
		Menstrual blood derived stem cells	Case report, mouse experiment。	It can help thicken the endometrium, but it is rarely used clinically. It has good safety, but it cannot be applied to patients with amenorrhea.	(52–55)
		ADSCs and ADRCs	Pilot Study, mouse experiment.	Intrauterine injection of ADSCs combined with subsequent hormone supplementation can significantly increase EMT, and ADRCs can improve endometrial thickness and embryo implantation in mice.	(56, 57)
		Umbilical cord mesenchymal stem cells	Case report, experiment on rats.	Repair the endometrium and improve fertility. Future applications need to worry about the risk of allogeneic sources.	(58, 59)
		Endometrial mesenchymal stem cells	Case report, experiment on rats, prospective studies.	Promote EMT growth while promoting live birth.	(62)
	Human embryonic stem cell	Human embryonic stem cell	Mouse experiment.	Experiments have shown that the endometrium can be repaired, but at the same time there is the possibility of non-directed differentiation to form tumors.	(63, 64)
Growth factor - granulocyte colony stimulating factor (G-CSF)		G-CSF	Animal experiments, retrospective cohort studies, prospective cohort studies.	It can increase endometrial thickness and improve pregnancy outcome.	(79–83)
Autologous platelet-rich plasma (PRP)		PRP	Retrospective study, prospective cohort study, randomized controlled study, *in vitro* experiment.	Increased endometrial thickness increased clinical pregnancy rate and live birth rate, but the influence was related to the concentration of PRP.	(90–94)
Complementary and alternative therapies		Chinese medicine, acupuncture, massage.	Meta-analysis, RCT.	Increase endometrial thickness, improve endometrial receptivity and increase pregnancy rate.	(95–99)

## 6 Conclusion

The endometrium plays a crucial role in embryo implantation. TE reduces endometrial tolerance, which in turn affects embryo implantation and development, leading to pregnancy failure. In ART, poor pregnancy outcomes in patients with TE are a major clinical challenge. In most patients, TE is associated with medical factors like previous uterine operations. This requires obstetricians and gynecologists to focus on the concept of primary endometrial prevention while researching and formulating clinical treatment plans, avoiding unnecessary uterine operations, and reducing the occurrence of medically-related TE at the source. TE can currently be treated through clinical measures ranging from hormone therapy, medication to improve endometrial blood flow, to regenerative medicine therapy to promote endometrial proliferation, traditional Chinese medicine alternative therapy and other adjuvant therapies. Nevertheless, pregnancy outcomes among patients with TE remain unsatisfactory. When the effect of single treatment is inadequate, a combination of multiple treatment methods has been tried; however, most therapies lack a large-sample evidence base. Thus, more high-quality large-sample clinical studies, with experimental designs, are needed to investigate these molecular mechanisms. We expect these to lead to methods that improve pregnancy outcomes among patients with TE, including increased pregnancy rate and LBR.

## Author contributions

YW: Writing – original draft, Investigation. ZT: Writing – original draft, Validation. XT: Writing – review & editing, Project administration, Funding acquisition.

## References

[1] Martins RS Oliani AH Oliani DV de Oliveira JM. Continuous endometrial volumetric analysis for endometrial receptivity assessment on assisted reproductive technology cycles. BMC Pregnancy Childbirth. (2020) 20:663. doi: 10.1186/s12884-020-03372-2 33143675 PMC7640386

[2] Leone FP Timmerman D Bourne T Valentin L Epstein E Goldstein SR. Terms, definitions and measurements to describe the sonographic features of the endometrium and intrauterine lesions: a consensus opinion from the International Endometrial Tumor Analysis (IETA) group. Ultrasound Obstet Gynecol. (2010) 35:103–12. doi: 10.1002/uog.7487 20014360

[3] Wu J Huang J Dong J Xiao X Li M Wang X. The thicker the endometrium, the better the neonatal outcomes? Hum Reprod Open. (2023) 2023. doi: 10.1093/hropen/hoad028 PMC10363027 37489142

[4] Liu KE Hartman M Hartman A Luo ZC Mahutte N. The impact of a thin endometrial lining on fresh and frozen–thaw IVF outcomes: an analysis of over 40 000 embryo transfers. Hum Reprod. (2018) 33:1883–8. doi: 10.1093/humrep/dey281 PMC6145412 30239738

[5] Mahutte N Hartman M Meng L Lanes A Luo Z-C Liu KE. Optimal endometrial thickness in fresh and frozen-thaw in *vitro* fertilization cycles: an analysis of live birth rates from 96,000 autologous embryo transfers. Fertil Steril.. (2022) 117:792–800. doi: 10.1016/j.fertnstert.2021.12.025 35109980

[6] Mahajan N Sharma S. The endometrium in assisted reproductive technology: How thin is thin? J Hum Reprod Sci. (2016) 9:3–8. doi: 10.4103/0974-1208.178632 27110071 PMC4817285

[7] Kasius A Smit JG Torrance HL Eijkemans MJ Mol BW Opmeer BC. Endometrial thickness and pregnancy rates after IVF: a systematic review and meta-analysis. Hum Reprod Update. (2014) 20:530–41. doi: 10.1093/humupd/dmu011 24664156

[8] Kumbak B Erden HF Tosun S Akbas H Ulug U Bahceci M. Outcome of assisted reproduction treatment in patients with endometrial thickness less than 7 mm. Reprod Biomedicine Online. (2009) 18:79–84. doi: 10.1016/S1472-6483(10)60428-2 19146773

[9] Lee D Jo JD Kim SK Jee BC Kim SH. The efficacy of intrauterine instillation of granulocyte colony-stimulating factor in infertile women with a thin endometrium: A pilot study. Clin Exp Reprod Med. (2016) 43:240–6. doi: 10.5653/cerm.2016.43.4.240 PMC5234289 28090464

[10] Mouhayar Y Sharara FI. G-CSF and stem cell therapy for the treatment of refractory thin lining in assisted reproductive technology. J Assist Reprod Genet. (2017) 34:831–7. doi: 10.1007/s10815-017-0922-6 PMC5476539 28405864

[11] Zhao J Zhang Q Li Y. The effect of endometrial thickness and pattern measured by ultrasonography on pregnancy outcomes during IVF-ET cycles. Reprod Biol Endocrinol. (2012) 10:100. doi: 10.1186/1477-7827-10-100 23190428 PMC3551825

[12] Kunicki M Lukaszuk K Woclawek-Potocka I Liss J Kulwikowska P Szczyptanska J. Evaluation of granulocyte colony-stimulating factor effects on treatment-resistant thin endometrium in women undergoing in *vitro* fertilization. BioMed Res Int. (2014) 2014:913235. doi: 10.1155/2014/913235 24693540 PMC3944906

[13] Liu KE Hartman M Hartman A. Management of thin endometrium in assisted reproduction: a clinical practice guideline from the Canadian Fertility and Andrology Society. Reprod BioMed Online. (2019) 39:49–62. doi: 10.1016/j.rbmo.2019.02.013 31029557

[14] Wang Y Lyu W Xu W Yu Y. Asherman syndrome in adenomyosis treated with uterine artery embolization: incidence predictive factors. Radiol Med. (2020) 125:437–43. doi: 10.1007/s11547-020-01136-8 32020527

[15] Miwa I Tamura H Takasaki A Yamagata Y Shimamura K Sugino N. Pathophysiologic features of "thin" endometrium. Fertil Steril. (2009) 91:998–1004. doi: 10.1016/j.fertnstert.2008.01.029 18328483

[16] Zheng Y Chen B Dai J Xu B Ai J Jin L. Thin endometrium is associated with higher risks of preterm birth and low birth weight after frozen single blastocyst transfer. Front Endocrinol (Lausanne). (2022) 13:1040140. doi: 10.3389/fendo.2022.1040140 36440225 PMC9685422

[17] Oron G Hiersch L Rona S Prag-Rosenberg R Sapir O Tuttnauer-Hamburger M. Endometrial thickness of less than 7.5 mm is associated with obstetric complications in fresh IVF cycles: a retrospective cohort study. Reprod BioMed Online. (2018) 37:341–8. doi: 10.1016/j.rbmo.2018.05.013 30146441

[18] Mouhayar Y Franasiak JM Sharara FI. Obstetrical complications of thin endometrium in assisted reproductive technologies: a systematic review. J Assist Reprod Genet. (2019) 36:607–11. doi: 10.1007/s10815-019-01407-y PMC6505009 30684071

[19] Cha J Sun X Dey SK. Mechanisms of implantation: strategies for successful pregnancy. Nat Med. (2012) 18:1754–67. doi: 10.1038/nm.3012 PMC6322836 23223073

[20] Shen MS Wang CW Chen CH Tzeng CR. New horizon on successful management for a woman with repeated implantation failure due to unresponsive thin endometrium: Use of extended estrogen supplementation. J Obstet Gynaecol Re. (2013) 39:1092–4. doi: 10.1111/j.1447-0756.2012.02070.x 23379608

[21] Song H Lim H Das SK Paria BC Dey SK. Dysregulation of EGF family of growth factors and COX-2 in the uterus during the preattachment and attachment reactions of the blastocyst with the luminal epithelium correlates with implantation failure in LIF-deficient mice. Mol Endocrinol. (2000) 14:1147–61. doi: 10.1210/mend.14.8.0498 10935540

[22] Stewart CL Kaspar P Brunet LJ Bhatt H Gadi I Kontgen F. Blastocyst implantation depends on maternal expression of leukaemia inhibitory factor. Nature. (1992) 359:76–9. doi: 10.1038/359076a0 1522892

[23] Amir W Micha B Ariel H Liat LG Jehoshua D Adrian S. Predicting factors for endometrial thickness during treatment with assisted reproductive technology. Fertil Steril. (2007) 87:799–804. doi: 10.1016/j.fertnstert.2006.11.002 17207799

[24] Liu SM Zhou YZ Wang HB Sun ZY Zhen JR Shen K. Factors associated with effectiveness of treatment and reproductive outcomes in patients with thin endometrium undergoing estrogen treatment. Chin Med J (Engl). (2015) 128:3173–7. doi: 10.4103/0366-6999.170258 PMC4794872 26612292

[25] Yi T Zhang X Gupta V Li L Zhong Q. Transdermal estrogen gel vs oral estrogen after hysteroscopy for intrauterine adhesion separation: A prospective randomized study. Front Endocrinol (Lausanne). (2023) 14:1066210. doi: 10.3389/fendo.2023.1066210 36967790 PMC10031037

[26] Almeida-Francia CC Keator CS Mah K Holden L Hergert C Slayden OD. Localization and hormonal regulation of endometrial matrix metalloproteinase-26 in the rhesus macaque. Hum Reprod. (2012) 27:1723–34. doi: 10.1093/humrep/des086 PMC3357194 22434853

[27] Hosseini Aghdam S Ghasemzadeh A Farzadi L Hamdi K Baradaran-Binazir M Nouri M. Growth hormone: A potential treatment of patients with refractory thin endometrium: A clinical trial study. Int J Fertil Steril. (2022) 16:251–5. doi: 10.22074/ijfs.2022.541389.1210 PMC9627013 36273309

[28] Xue-Mei W Hong J Wen-Xiang Z Yang L. The effects of growth hormone on clinical outcomes after frozen-thawed embryo transfer. Int J Gynaecol Obstet. (2016) 133:347–50. doi: 10.1016/j.ijgo.2015.10.020 27101995

[29] Shang Y Wu M He R Ye Y Sun X. Administration of growth hormone improves endometrial function in women undergoing in *vitro* fertilization: a systematic review and meta-analysis. Hum Reprod Update. (2022) 28:838–57. doi: 10.1093/humupd/dmac028 35641113

[30] Fogle RH Li A Paulson RJ. Modulation of HOXA10 and other markers of endometrial receptivity by age and human chorionic gonadotropin in an endometrial explant model. Fertil Steril. (2010) 93:1255–9. doi: 10.1016/j.fertnstert.2008.11.002 19131054

[31] Liu Y Ma L Zhu M Yin H Yan H Shi M. STROBE-GnRHa pretreatment in frozen-embryo transfer cycles improves clinical outcomes for patients with persistent thin endometrium: A case-control study. Med (Baltimore). (2022) 101:e29928. doi: 10.1097/MD.0000000000029928 PMC9351881 35945767

[32] Wang P Yang H Chen Z Chen Y Jin C Yu R. Agonist long protocol improves outcomes of vitrified-warmed embryo transfer in repeatedly thin endometrium. Reprod BioMed Online. (2023) 46:527–35. doi: 10.1016/j.rbmo.2022.12.003 36604214

[33] Zhao D Xie R Li X. Comparison of pregnancy outcome after fresh embryo transfer between GnRH antagonist and GnRH agonist regimens in patients with thin endometrium. Front Med. (2023) 10:1071014. doi: 10.3389/fmed.2023.1071014 PMC9892192 36744125

[34] Shi Q Huang C Liu J Li Y Kong N Mei J. Hormone replacement therapy alone or in combination with tamoxifen in women with thin endometrium undergoing frozen-thawed embryo transfer: A retrospective study. Front Endocrinol. (2023) 14:1102706. doi: 10.3389/fendo.2023.1102706 PMC10014925 36936160

[35] Sharma S Rani G Bose G Saha I Bathwal S Chakravarty BN. Tamoxifen is Better than Low-Dose Clomiphene or Gonadotropins in Women with Thin Endometrium (<7 mm) after Clomiphene in Intrauterine Insemination Cycles: A Prospective Study. J Hum Reprod Sci. (2018) 11:34–9. doi: 10.4103/jhrs.JHRS_9_17 PMC5892102 29681714

[36] Lambers MJ Hoozemans DA Schats R Homburg R Lambalk CB Hompes PGA. Low-dose aspirin in non-tubal IVF patients with previous failed conception: a prospective randomized double-blind placebo-controlled trial. Fertil Steril. (2009) 92:923–9. doi: 10.1016/j.fertnstert.2008.07.1759 18973893

[37] Hsieh YY Tsai HD Chang CC Lo HY Chen CL. Low-dose aspirin for infertile women with thin endometrium receiving intrauterine insemination: a prospective, randomized study. J Assist Reprod Genet. (2000) 17:174–7. doi: 10.1023/a:1009474307376 PMC3455661 10911579

[38] Chen Y Liu L Luo Y Chen M Huan Y Fang R. Effects of aspirin and intrauterine balloon on endometrial repair and reproductive prognosis in patients with severe intrauterine adhesion: A prospective cohort study. BioMed Res Int. (2017) 2017:8526104. doi: 10.1155/2017/8526104 28251159 PMC5303840

[39] Li X Luan T Zhao C Zhang M Dong L Su Y. Effect of sildenafil citrate on treatment of infertility in women with a thin endometrium: a systematic review and meta-analysis. J Int Med Res. (2020) 48:300060520969584. doi: 10.1177/0300060520969584 33176524 PMC7673063

[40] Moini A Zafarani F Jahangiri N Jahanian Sadatmahalleh SH Sadeghi M Chehrazi M. The effect of vaginal sildenafil on the outcome of assisted reproductive technology cycles in patients with repeated implantation failures: A randomized placebo-controlled trial. Int J Fertil Steril. (2020) 13:289–95. doi: 10.22074/ijfs.2020.5681 PMC6875854 31710189

[41] Kwon KH Shin KS Yeon SH Kwon DG. Application of botulinum toxin in maxillofacial field: Part III. Ancillary treatment for maxillofacial surgery and summary. Maxillofac Plast Reconstr Surg. (2019) 41:45. doi: 10.1186/s40902-019-0226-0 31709199 PMC6813409

[42] Sundaram H Liew S Signorini M Vieira Braz A Fagien S Swift A. Global aesthetics consensus: hyaluronic acid fillers and botulinum toxin type A-recommendations for combined treatment and optimizing outcomes in diverse patient populations. Plast Reconstr Surg. (2016) 137:1410–23. doi: 10.1097/PRS.0000000000002119 PMC5242215 27119917

[43] Gugerell A Kober J Schmid M Buchberger E Kamolz LP Keck M. Botulinum toxin A: dose-dependent effect on reepithelialization and angiogenesis. Plast Reconstr Surg Glob Open. (2016) 4:e837. doi: 10.1097/GOX.0000000000000852 27622105 PMC5010328

[44] Koo HS Yoon MJ Hong SH Ahn J Cha H Lee D. Non-invasive intrauterine administration of botulinum toxin A enhances endometrial angiogenesis and improves the rates of embryo implantation. Reprod Sci. (2021) 28:1671–87. doi: 10.1007/s43032-021-00496-4 PMC8144131 33650094

[45] Lee D Ahn J Koo HS Kang YJ. Intrauterine botulinum toxin A administration promotes endometrial regeneration mediated by IGFBP3-dependent OPN proteolytic cleavage in thin endometrium. Cell Mol Life Sci. (2023) 80:26. doi: 10.1007/s00018-022-04684-6 36602651 PMC9816300

[46] Krief F Simon C Goldstein R Ellenberg LP Ledee N. Efficacy of tocopherol and pentoxifylline combined therapy for women undergoing assisted reproductive treatment with poor endometrial development: a retrospective cohort study on 143 patients. Hum Fertil (Camb). (2021) 24:367–75. doi: 10.1080/14647273.2019.1673906 31597488

[47] Lee YJ Yi KW. Bone marrow-derived stem cells contribute to regeneration of the endometrium. Clin Exp Reprod Med. (2018) 45:149–53. doi: 10.5653/cerm.2018.45.4.149 PMC6277671 30538944

[48] Patel NH Jadeja YD Patel NH Patel MN Bhadarka HK Chudasama PN. Birth of a healthy infant after bone marrow-derived cell therapy. Clin Exp Reprod Med. (2021) 48:268–72. doi: 10.5653/cerm.2020.04252 PMC8421658 34488290

[49] Cervello I Gil-Sanchis C Santamaria X Cabanillas S Diaz A Faus A. Human CD133(+) bone marrow-derived stem cells promote endometrial proliferation in a murine model of Asherman syndrome. Fertil Steril. (2015) 104:1552–60.e1-3. doi: 10.1016/j.fertnstert.2015.08.032 26384164

[50] Wang G Ren CE Jiang J. Effects of bone marrow mesenchymal stem cells on repair and receptivity of damaged endometrium in rats. J Obstet Gynaecol Re. (2021) 47:3223–31. doi: 10.1111/jog.14888 34184363

[51] Tapil’skaya NI Ob’edkova KV Gzgzyan AM Bespalova ON. Cell-based therapy in thin endometrium syndrome. Russian Military Med Acad Rep. (2023) 42:23–8. doi: 10.17816/rmmar109077

[52] Tan J Li P Wang Q Li Y Li X Zhao D. Autologous menstrual blood-derived stromal cells transplantation for severe Asherman's syndrome. Hum Reprod. (2016) 31:2723–9. doi: 10.1093/humrep/dew235 27664218

[53] Zhao M Chi F Zhang T Teng X Li K. Human menstrual blood-derived mesenchymal stem cells regulation of the EGF/Ras p21 pathway as a potential therapeutic target for thin endometrium. Ann Trans Med. (2021) 9:1476–. doi: 10.21037/atm-21-4652 PMC8506758 34734028

[54] Zheng SX Wang J Wang XL Ali A Wu LM Liu YS. Feasibility analysis of treating severe intrauterine adhesions by transplanting menstrual blood-derived stem cells. Int J Mol Med. (2018) 41:2201–12. doi: 10.3892/ijmm.2018.3415 29393381

[55] Chen K Wang H Zhao X Wang J Jin Q Tong X. A novel method to repair thin endometrium and restore fertility based on menstruation-derived stem cell. Reprod Sci. (2024). doi: 10.1007/s43032-024-01458-2 PMC11111544 38294669

[56] Lee SY Shin JE Kwon H Choi DH Kim JH. Effect of autologous adipose-derived stromal vascular fraction transplantation on endometrial regeneration in patients of asherman's syndrome: a pilot study. Reprod Sci. (2020) 27:561–8. doi: 10.1007/s43032-019-00055-y 32046396

[57] Yotsumoto F Iwaguro H Harada Y Sobajima S Suwabe T Miyamoto S. Adipose tissue-derived regenerative cells improve implantation of fertilized eggs in thin endometrium. Regener Med. (2020) 15:1891–904. doi: 10.2217/rme-2020-0037 32698666

[58] Zhang L Li Y Guan CY Tian S Lv XD Li JH. Therapeutic effect of human umbilical cord-derived mesenchymal stem cells on injured rat endometrium during its chronic phase. Stem Cell Res Ther. (2018) 9:36. doi: 10.1186/s13287-018-0777-5 29433563 PMC5810045

[59] Cao Y Sun H Zhu H Zhu X Tang X Yan G. Allogeneic cell therapy using umbilical cord MSCs on collagen scaffolds for patients with recurrent uterine adhesion: a phase I clinical trial. Stem Cell Res Ther. (2018) 9. doi: 10.1186/s13287-018-0904-3 PMC6042450 29996892

[60] Al-Lamee H Hill CJ Turner F Phan T Drakeley AJ Hapangama DK. The role of endometrial stem/progenitor cells in recurrent reproductive failure. J Personalized Med. (2022) 12. doi: 10.3390/jpm12050775 PMC9143189 35629197

[61] Fayazi M Salehnia M Ziaei S. Differentiation of human CD146-positive endometrial stem cells to adipogenic-, osteogenic-, neural progenitor-, and glial-like cells. In Vitro Cell Dev Biol - Anim. (2014) 51:408–14. doi: 10.1007/s11626-014-9842-2 25515247

[62] Sapozhak IM Gubar capital OC Rodnichenko AE Zlatska AV. Application of autologous endometrial mesenchymal stromal/stem cells increases thin endometrium receptivity: a case report. J Med Case Rep. (2020) 14:190. doi: 10.1186/s13256-020-02515-5 33066810 PMC7568420

[63] Song T Zhao X Sun H Li X Lin N Ding L. Regeneration of uterine horns in rats using collagen scaffolds loaded with human embryonic stem cell-derived endometrium-like cells. Tissue Eng Part A. (2015) 21:353–61. doi: 10.1089/ten.TEA.2014.0052 PMC4292859 25097004

[64] Wang YH QJ Lv YF Jiang YP. Preliminary study of embryonic stem cells transplanted into the injuried endometrium of mouse. J Int Reprod Health/Fam Plan. (2012) 31:434–5+48+503. doi: 10.3969/j.issn.1674-1889.2012.06.002

[65] Jin X Xin L Zhang S. Effect of extracellular vesicles on improving endometrial receptivity and the research status. Chin J Pract Gynecology Obstetrics. (2022) 38:895–9. doi: 10.19538/j.fk2022090108

[66] Kowalczyk A Wrzecińska M Czerniawska-Piątkowska E Kupczyński R. Exosomes – Spectacular role in reproduction. Biomedicine Pharmacotherapy. (2022) 148. doi: 10.1016/j.biopha.2022.112752 35220028

[67] Cheng Q Shi X Han M Smbatyan G Lenz H-J Zhang Y. Reprogramming exosomes as nanoscale controllers of cellular immunity. J Am Chem Soc. (2018) 140:16413–7. doi: 10.1021/jacs.8b10047 PMC6469991 30452238

[68] He C Zheng S Luo Y Wang B. Exosome theranostics: biology and translational medicine. Theranostics. (2018) 8:237–55. doi: 10.7150/thno.21945 PMC5743472 29290805

[69] Valadi H Ekström K Bossios A Sjöstrand M Lee JJ Lötvall JO. Exosome-mediated transfer of mRNAs and microRNAs is a novel mechanism of genetic exchange between cells. Nat Cell Biol. (2007) 9:654–U72. doi: 10.1038/ncb1596 17486113

[70] Jiang N-X Li X-L. The complicated effects of extracellular vesicles and their cargos on embryo implantation. Front Endocrinol. (2021) 12:681266. doi: 10.3389/fendo.2021.681266 PMC8213030 34149619

[71] Liao Z Liu C Wang L Sui C Zhang H. Therapeutic role of mesenchymal stem cell-derived extracellular vesicles in female reproductive diseases. Front Endocrinol (Lausanne). (2021) 12:665645. doi: 10.3389/fendo.2021.665645 34248842 PMC8261239

[72] Zhao Y Tao M Wei M Du S Wang H Wang X. Mesenchymal stem cells derived exosomal miR-323-3p promotes proliferation and inhibits apoptosis of cumulus cells in polycystic ovary syndrome (PCOS). Artif Cells Nanomedicine Biotechnol. (2019) 47:3804–13. doi: 10.1080/21691401.2019.1669619 31549864

[73] Chang Y Liu Y Li X. Exosomes derived from human umbilical cord mesenchymal stem cells promote proliferation of endometrial stromal cell. Fertility Sterility. (2020), 114(3): E525–E525. doi: 10.1016/j.fertnstert.2020.09.035

[74] Ebrahim N Mostafa O El Dosoky RE Ahmed IA Saad AS Mostafa A. Human mesenchymal stem cell-derived extracellular vesicles/estrogen combined therapy safely ameliorates experimentally induced intrauterine adhesions in a female rat model. Stem Cell Res Ther. (2018) 9. doi: 10.1186/s13287-018-0924-z PMC6027762 29954457

[75] Yao Y Chen R Wang G Zhang Y Liu F. Exosomes derived from mesenchymal stem cells reverse EMT via TGF-β1/Smad pathway and promote repair of damaged endometrium. Stem Cell Res Ther. (2019) 10. doi: 10.1186/s13287-019-1332-8 PMC6664513 31358049

[76] Perrini C Strillacci MG Bagnato A Esposti P Marini MG Corradetti B. Microvesicles secreted from equine amniotic-derived cells and their potential role in reducing inflammation in endometrial cells in an in-vitro model. Stem Cell Res Ther. (2016) 7:169. doi: 10.1186/s13287-016-0429-6 27863532 PMC5114748

[77] Blázquez R Sánchez-Margallo FM Álvarez V Matilla E Hernández N Marinaro F. Murine embryos exposed to human endometrial MSCs-derived extracellular vesicles exhibit higher VEGF/PDGF AA release, increased blastomere count and hatching rates. PloS One. (2018) 13:e0196080. doi: 10.1371/journal.pone.0196080 29684038 PMC5912768

[78] Xin L Lin X Zhou F Li C Wang X Yu H. A scaffold laden with mesenchymal stem cell-derived exosomes for promoting endometrium regeneration and fertility restoration through macrophage immunomodulation. Acta Biomaterialia. (2020) 113:252–66. doi: 10.1016/j.actbio.2020.06.029 32574858

[79] Isik G Oktem M Guler I Oktem E Ozogul C Saribas S. The impact of granulocyte colony-stimulating factor (G-CSF) on thin endometrium of an animal model with rats. Gynecol Endocrinol. (2021) 37:438–45. doi: 10.1080/09513590.2020.1786508 32611261

[80] Xie Y Tian Z Qi Q Li Z Bi Y Qin A. The therapeutic effects and underlying mechanisms of the intrauterine perfusion of granulocyte colony-stimulating factor on a thin-endometrium rat model. Life Sci. (2020) 260:118439. doi: 10.1016/j.lfs.2020.118439 32950574

[81] Gleicher N Vidali A Barad DH. Successful treatment of unresponsive thin endometrium. Fertil Steril. (2011) 95:2123.e13–7. doi: 10.1016/j.fertnstert.2011.01.143 21324451

[82] Lian R Wang X Lin R Zeng H Zeng Y Liu S. Evaluation of granulocyte colony-stimulating factor on the treatment of thin endometrium during frozen-thawed embryo transfer cycles: a retrospective cohort study. Gynecol Endocrinol. (2020) 36:370–4. doi: 10.1080/09513590.2019.1658187 31464150

[83] Jiang L Xu X Cao Z Yang N Wang S Wang L. Comparison of frozen embryo transfer outcomes between uterine infusion of granulocyte colony-stimulating factor and growth hormone application in patients with thin endometrium: A retrospective study. Front Endocrinol (Lausanne). (2021) 12:725202. doi: 10.3389/fendo.2021.725202 35027908 PMC8750567

[84] Dohan DM Choukroun J Diss A Dohan SL Dohan AJ Mouhyi J. Platelet-rich fibrin (PRF): a second-generation platelet concentrate. Part I: technological concepts and evolution. Oral Surg Oral Med Oral Pathol Oral Radiol Endod. (2006) 101:e37–44. doi: 10.1016/j.tripleo.2005.07.008 16504849

[85] Dohan Ehrenfest DM de Peppo GM Doglioli P Sammartino G. Slow release of growth factors and thrombospondin-1 in Choukroun's platelet-rich fibrin (PRF): a gold standard to achieve for all surgical platelet concentrates technologies. Growth Factors. (2009) 27:63–9. doi: 10.1080/08977190802636713 19089687

[86] Matras H. Effect of various fibrin preparations on reimplantations in the rat skin. Osterr Z Stomatol. (1970) 67:338–59. 4917644

[87] Conde Montero E Fernandez Santos ME Suarez Fernandez R. Platelet-rich plasma: applications in dermatology. Actas Dermosifiliogr. (2015) 106:104–11. doi: 10.1016/j.ad.2013.12.021 24795093

[88] Everts PA van Erp A DeSimone A Cohen DS Gardner RD. Platelet rich plasma in orthopedic surgical medicine. Platelets. (2021) 32:163–74. doi: 10.1080/09537104.2020.1869717 33400591

[89] Justicz N Derakhshan A Chen JX Lee LN. Platelet-rich plasma for hair restoration. Facial Plast Surg Clin North Am. (2020) 28:181–7. doi: 10.1016/j.fsc.2020.01.009 32312505

[90] Chang Y Li J Li X Yang X Liang X. Platelet-rich plasma administration has benefit for infertile women with thin endometrium in frozen blastocyst-stage embryos transfer program. Fertility Sterility. (2017) 108:E77–E. doi: 10.1016/j.fertnstert.2017.07.243

[91] Coksuer H Akdemir Y Ulas Barut M. Improved in *vitro* fertilization success and pregnancy outcome with autologous platelet-rich plasma treatment in unexplained infertility patients that had repeated implantation failure history. Gynecol Endocrinol. (2019) 35:815–8. doi: 10.1080/09513590.2019.1597344 30966843

[92] Pandey D Bajaj B Kapoor G Bharti R. Intrauterine instillation of autologous platelet-rich plasma in infertile females with thin endometrium undergoing intrauterine insemination: an open-label randomized controlled trial. AJOG Global Rep. (2023) 3. doi: 10.1016/j.xagr.2023.100172 PMC10027558 36960130

[93] Wang X Liu L Mou S Zhao H Fang J Xiang Y. Investigation of platelet-rich plasma in increasing proliferation and migration of endometrial mesenchymal stem cells and improving pregnancy outcome of patients with thin endometrium. J Cell Biochem. (2019) 120:7403–11. doi: 10.1002/jcb.28014 30506864

[94] Yang Y LM Li JP Zu ZY Wu L Zhang K. The effects of peripheral venous blood and umbilical vein blood-derived platelet-rich plasma on the proliferation of endometrial stromal cells. Prog Obstet Gynecol. (2020) 29:513–6+21. doi: 10.13283/j.cnki.xdfckjz.2020.07.031

[95] Zhang X Chen Y Zhao R Chen Y Xing P Xu W. The clinical efficacy of Jinfeng pill in the treatment of thin endometrial infertility: a systematic review and meta-analysis. Ann Palliat Med. (2021) 10:12529–37. doi: 10.21037/apm-21-3354 35016454

[96] Zheng J Tan Y. [Effects of Dingkun Dan combined with Western medicine on endometrial beta-catenin, VEGF and MMP-9 mRNA expression in rats with multiple lesions]. Zhongguo Zhong Yao Za Zhi. (2020) 45:3713–8. doi: 10.19540/j.cnki.cjcmm.20200227.402 32893563

[97] Yin XD Xue XO Wang JS Yang W He JQ. Effect of Bushen Huoxue recipe on women with thin endometrial ovulation disorder and a rat model of thin endometrium resulted from kidney deficiency-related blood stasis. Gynecol Endocrinol. (2021) 37:433–7. doi: 10.1080/09513590.2020.1781079 32584196

[98] Qi Y Wang X Hou S Wu Z Xu X Pang C. Intracavitary physiotherapy combined with acupuncture mediated AMPK/mTOR signalling to improve endometrial receptivity in patients with thin endometrium. Eur J Obstet Gynecol Reprod Biol. (2022) 277:32–41. doi: 10.1016/j.ejogrb.2022.08.002 35987076

[99] Shen L. Effects of pelvic floor muscle massage on the pregnancy outcome of frozen embryo transfer in patients with thin endometrium. Comput Math Methods Med. (2022) 2022:2803363. doi: 10.1155/2022/2803363 35813410 PMC9259337

